# How the COVID-19 Epidemic Affected Prehospital Emergency Medical Services in Tehran, Iran

**DOI:** 10.5811/westjem.2020.8.48679

**Published:** 2020-09-25

**Authors:** Peyman Saberian, Joseph L. Conovaloff, Elnaz Vahidi, Parisa Hasani-Sharamin, Pir-Hossein Kolivand

**Affiliations:** * Tehran University of Medical Sciences, Prehospital and Hospital Emergency Research Center, Tehran, Iran; † Tehran University of Medical Sciences, Department of Anesthesiology, Imam Khomeini Hospital Complex, Tehran, Iran; ‡ UC Irvine School of Medicine, Department of Emergency Medicine, Orange, CA, United States of America; § Tehran University of Medical Sciences, Shariati Hospital, Department of Emergency Medicine, Tehran, Iran; ¶ Tehran Emergency Medical Service Center, Tehran, Iran; || Iranian Emergency Medical Service Organization, Tehran, Iran; # Khatamol Anbia Hospital, Shefa Neuroscience Research Center, Tehran, Iran

## Abstract

**Introduction:**

Coronavirus disease 2019 (COVID-19) has substantially impacted the healthcare delivery system in Tehran, Iran. The country’s first confirmed positive test for severe acute respiratory syndrome coronavirus-2 (SARS-CoV-2) was on February 18, 2020. Since then, the number of cases has steadily increased in Iran and worldwide. Emergency medical services (EMS) quickly adapted its operations to accommodate a greater number of patients, and it worked to decrease the risk of COVID-19 spread among EMS personnel, given the disease’s high transmissibility.

**Methods:**

We evaluated the chief complaint as well as the pattern and number of EMS calls and dispatches during the 28-day intervals before and after the February 18, 2020, COVID-19 outbreak in Iran.

**Results:**

EMS calls increased from 355,241 in the pre-outbreak period to 1,589,346 in the post-outbreak period, a 347% increase (p<0.001). EMS dispatches rose more modestly from 82,282 to 99,926, a 21% increase (p<0.001). The average time on telephone hold decreased from 10.6 ± 12.7 seconds pre-outbreak to 9.8 ± 11.8 seconds post-outbreak, a 7% decrease (p<0.001). The average length of call also decreased from 1.32 ± 1.42 minutes pre-outbreak to 1.06 ± 1.28 minutes post-outbreak, a 20% decrease (p<0.001). The highest number of daily dispatches occurred during the second and third weeks of the four-week post-outbreak period, peaking at 4557 dispatches/day. After the first reported case of SARS-CoV-2, there were significant increases in chief complaints of fever (211% increase, p<0.001) and respiratory symptoms (245% increase, p<0.001).

**Conclusion:**

The number of EMS calls and dispatches in Tehran increased 347% and 20%, respectively, after the outbreak of COVID-19. Despite this, the time on hold for EMS response decreased. The Tehran EMS system accomplished this by increasing personnel hours, expanding call-center resources, and implementing COVID-19-specific training.

## INTRODUCTION

Coronavirus disease 2019 (COVID-19), caused by severe acute respiratory syndrome coronavirus-2 (SARS-CoV-2), was first discovered in humans in Wuhan China, late last year.^1^ It has presented a unique challenge to a healthcare delivery system not prepared for major healthcare catastrophes. Without prior crisis management plans in place, many hospitals have faced a lack of medical supplies, increased patient load, and an exhausted medical staff. The current pandemic highlights these deficits in disaster preparedness and the importance of developing a systematic approach to deal with future healthcare crises.

On February 18, 2020, Iran’s first positive test for SARS-CoV-2 was reported. One day later, Iran’s Ministry of Health confirmed the beginning of the outbreak. The number of cases of COVID-19 in Iran has since increased substantially. On March 11, 2020, the World Health Organization (WHO) designated COVID-19 a pandemic. Globally, as of September 27, 2020, there were over 32.7 million confirmed cases of COVID-19 with 991,224 deaths reported to WHO. In Iran there were 443,086 confirmed cases of COVID-19 and a death toll of 25,394.^2^

Prehospital and hospital services were at first overwhelmed by fever and respiratory complaints in patients suspected of having COVID-19, which required the emergency medical services (EMS) to change its operating procedures. The EMS Organization in Tehran (EMS Tehran) created the Advanced Surveillance System of Coronavirus Committee. EMS Tehran increased the number of personnel working in order to adequately respond to the increase in patient numbers, and gave formal training to its employees to screen for and diagnose COVID-19. Employees were given more personal protective equipment (PPE) and essential supplies so that they could adequately care for patients. EMS Tehran also greatly extended its operations, limited the amount of time off for its employees, and added coronavirus-consulting phone lines to answer patient questions. The goal of this study was to determine the effects of COVID-19 on the workload of EMS Tehran and the associated changes to patient presentation on EMS arrival.

## METHODS

We collected EMS data including the number of calls and dispatches, patient complaints, and vital signs before and after the beginning of the COVID-19 outbreak in Tehran, a city with a population of 8.7 million. We divided our study into two 28-day periods, defining the pre-outbreak period as January 21–February 17, 2020, and the post-outbreak period as February 18–March 16, 2020. This study was approved by the ethics committee of Tehran University of Medical Sciences.

**Population Health Research Capsule**

What do we already know about this issue?

*Emergency medical services (EMS) has been forced to change protocols to maximize employee work hours and minimize waste of personal protective equipment*.

What was the research question?

*We sought to determine the effects of the COVID-19 pandemic on patient presentation and the workload of EMS Tehran*.

What was the major finding of the study?

*The number of dispatches, calls, and patients with fever and respiratory complaints increased after the outbreak in Tehran*.

How does this improve population health?

*By understanding the effects of the COVID-19 pandemic on EMS workload, we can optimize our policies to improve our response to future pandemics*.

### EMS in Iran

EMS Iran is an affiliate of Iran’s Ministry of Health.^3^ It oversees multiple departments including operations, administrative, financial, medical emergency communications, dispatch, quality control, method improvement, education, and research. Patients call “115” and speak with the emergency medical dispatcher (EMD) who takes a history and the caller’s address. The EMD gives this information to a nearby unit if a dispatch is deemed necessary. Emergency medical technicians (EMTs) evaluate the patient at the scene and may consult a physician in the dispatch center to determine whether the patient needs transport to a hospital. The EMTs then coordinate with the hospitals prior to arrival. There are 216 ambulance bases in Tehran, most with one ambulance and one motorcycle ambulance. The motorcycle ambulance is driven by one EMT to scenes where transport is not predicted to be necessary based on the dispatch call. The EMT may perform limited medical care. A few bases have two ambulances, and a few bases have an ambulance bus, which is used for multiple casualties when air transport is limited. All stations are managed by one dispatch center. There are 118 hospitals in Tehran, including 49 publicly run, 55 privately run, and 14 government run for the armed forces.

All EMDs are nurses with bachelor degrees, and EMTs have degrees in nursing, anesthesiology, or medical emergency. EMTs have different ranks including basic, intermediate, and paramedic, and the EMD takes this into account for a tiered response to calls. EMS personnel receive 60–200 hours of general training, and there are additional monthly in-service trainings. Since the COVID-19 outbreak, EMS Iran has also used virtual trainings including lectures and webinars.

### Changes to EMS workflow in Tehran

Changes to EMS workflow included adding “distance shifts” for EMDs working for the emergency communication (dispatch) center. These distance shifts occurred from employee homes to promote social distancing. Supervisors also began to answer dispatch calls. The number of dispatchers receiving calls at any time increased 140% from 20–24 to 36–48. We added 50 ambulances to the existing fleet, a 20% increase, to respond to calls. These added missions were staffed by base officials who did not routinely go on missions.

The EMS communication center and operating units began to ask COVID-19 screening questions to all patients, to identify patients with COVID-19 associated symptoms or a recent travel history to China. All employees were given formal training in recognizing and diagnosing COVID-19. EMS Tehran increased the amounts of PPE and essential medical supplies available for dispatches and reduced the number of personnel involved in each dispatch. These measures led to an adequate supply of PPE throughout the outbreak.

Volunteers ran PPE donation drives, and we received international donations as well. For dispatches involving patients suspected or confirmed to have COVID-19, all EMTs directly interacting with the patient wore “full” PPE including a gown, face shield, surgical mask, and gloves. This usually involved only one EMT to help conserve PPE. The other EMTs involved in the dispatch wore surgical masks and gloves. All PPE was thrown away after every dispatch, and the entire ambulance was subsequently washed and cleaned with disinfectants. Given the shortage of N95 masks, we disinfected and reused elastomeric masks.

Volunteer physicians and nurses with emergency medicine experience answered the general public’s questions on a different phone line. If they deemed medical attention was needed, they would connect the call to the dispatch center. This line received an average of 18,000 calls per day.

There was no change to the number of personnel performing dispatches. However, they did have increased overtime hours, reduced break time during shifts, and reduced number of hours between shifts. Before the COVID-19 outbreak, EMS personnel routinely worked 24-hour shifts and had 48 hours off between shifts. Time off between shifts decreased to 24 hours during the outbreak, resulting in an effective 50% increase in staff-hours.

At the beginning of the outbreak, Iran’s Ministry of Health designated 10 hospitals to treat COVID-19 patients. These hospitals saw the majority of suspected cases and also received transfers after coordination with the central operations guidance headquarters. EMS worked to transport patients with or suspected to have COVID-19 to these hospitals. Three to four percent of all dispatches were inter-hospital transfers.

### Data Analysis

We analyzed data using SPSS V.22 software (IBM Corporation, Armonk, NY). We verified normality with the Kolmogorov–Smirnov test. Data are presented as the mean ± SD or median with interquartile ranges as suitable. We used chi-square and Fisher’s exact tests to compare proportions of qualitative variables. Student’s t-test was used for parametric quantitative variables, and the Mann-Whitney U test was used for nonparametric quantitative variables. The level of significance was <0.05.

## RESULTS

**Figure 1:**
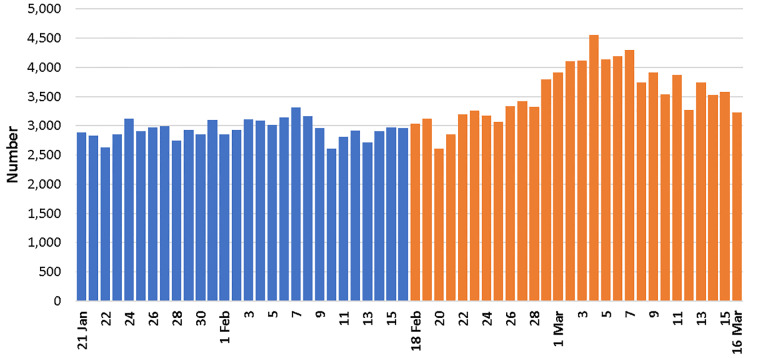
The number of emergency medical services dispatches before and after the beginning of the COVID-19 outbreak.

During the 56 days of the study, there were 182,208 EMS dispatches. The average number of daily dispatches in the pre- and post-outbreak periods was 2939 and 3569, respectively, a 21% increase (Table 1). The highest number of daily dispatches occurred on March 4, 2020, with 4557 dispatches (Figure 1). The number of daily dispatches during the second and third weeks of the post-outbreak period was consistently near 4000.

**Figure 2:**
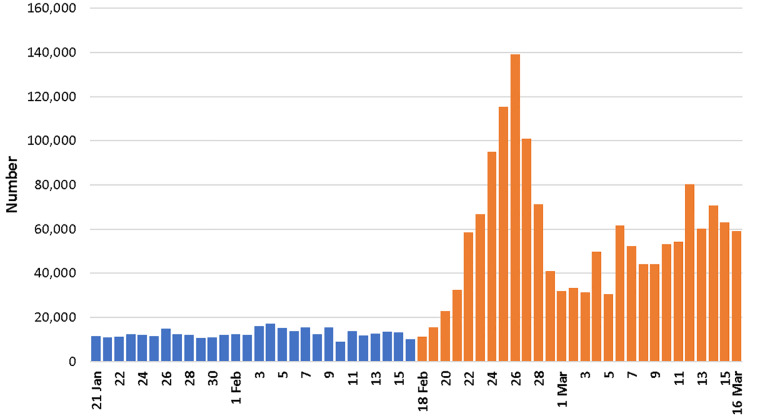
The number of emergency medical services phone calls before and after the beginning of the COVID-19 outbreak.

There was a substantially higher number of EMS phone calls during the post-outbreak period compared to the pre-outbreak period (Figure 2). We received 1,944,587 EMS calls, with a daily average of 12,687 EMS calls during the pre-outbreak period and 56,762 during the post-outbreak period (Table 1), a 347% increase (p<0.001). Phone call duration decreased from 1.3 ± 1.4 minutes (mean ± standard deviation) to 1.1 ± 1.3 minutes, a 20% decrease (p<0.001). The time waiting on hold decreased from 10.6 ± 12.7 seconds in the pre-outbreak period to 9.8 ± 11.8 seconds in the post-outbreak period, an 8% decrease (p<0.001). Peak time of day for phone calls to EMS occurred at 2 pm in the pre-outbreak period but at 10 pm in the post-outbreak period. Despite this trend, the peak time of day for dispatches occurred from 8 pm to 11 pm, which did not change between the pre- and post-outbreak periods.

We evaluated patient complaints and initial diagnoses as registered by EMTs. Fever and respiratory complaints were significantly more prevalent in the post-outbreak period, with a 211% and 245% increase, respectively.

## DISCUSSION

EMS is a crucial component of the healthcare delivery system and benefits from adapting its protocols during periods of high call volumes.^4^–^6^ Importantly, improving outpatient care can reduce the emergency department (ED) patient census and reduce the risk of overwhelming hospitals with limited resources and staff. Having fewer patients in the hospital further helps to avoid unnecessary transmission of SARS-CoV-2.

To improve EMS response during pandemics, the United States National EMS Advisory Council proposed having more fully developed prehospital triage algorithms, an auto-answer and caller deferral system for non-emergency situations, and alternate shift structures.^7^,^8^ Additionally, the council advocated for transporting patients to the closest hospital during high call periods and for minimizing the number of staff participating in each mission. During a pandemic, it is especially important to divert non-emergent patients to maximize EMS and hospital resources as well as to decrease the rate of disease transmission.^9^–^11^ Applying a triage and classification system can reduce the number of EMS responses, transports, and ED visits without adversely affecting patient outcomes.^12^,^13^ We used these advisories to our EMS response in Tehran.

We found that the number of calls to EMS and the number of EMS dispatches were both significantly increased during the post-outbreak period (Table 1). Chief complaints associated with COVID-19 were more prevalent during the post-outbreak period (Table 2). The increase in total EMS calls and EMS dispatches (Table 1), largely resulting from the increase in prevalence of chief complaints associated with COVID-19 (Table 2), demonstrates the significant impact COVID-19 had on the EMS system in Tehran. The number of traumas and motor vehicle accidents decreased during the outbreak period, which correlates with people staying home during the outbreak with quarantine precautions.

EMS increased the number of personnel responding to these calls and shortened their conversation times, which likely caused the call waiting time to decrease in the post-outbreak period, despite handling 4.5 times the number of EMS calls.

The EMS management center approved specific crisis management plans during the outbreak. The number of dispatch personnel receiving calls during each shift increased from 20–24 to 36–48 people, up to a 140% increase. A team of volunteer physicians and nurses responded to a different phone line to answer the general public’s questions regarding COVID-19, which helped to limit the number of calls to the dispatch phone line. This hotline had an average of 18,000 calls per day.

**Figure 3:**
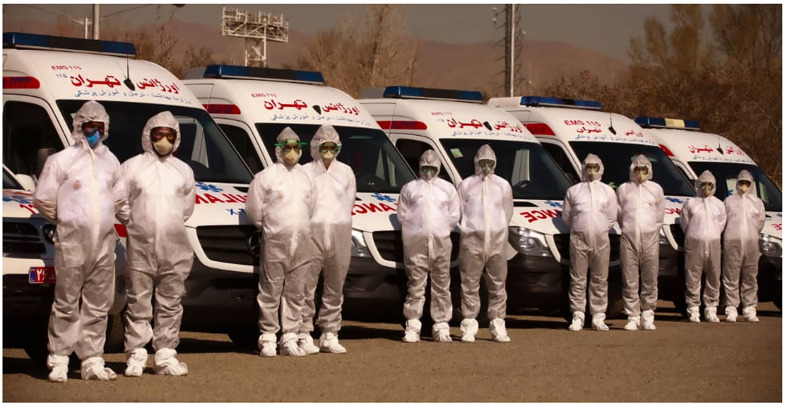
Iranian emergency medical services personnel during the COVID-19 epidemic.

EMS personnel wore gowns, overalls, face shields, surgical masks and gloves (Figure 3). Out of 118 total hospitals in Tehran, 10 were designated to take care of COVID-19 patients. Transferring patients to these hospitals was a priority. All employees of EMS Tehran used “How to Deal with COVID-19” guidelines created by Iran’s Ministry of Health, which outlined standard operating procedures during the outbreak.

EMS was able to respond appropriately to the increase in the number of calls and the increase in the number of patients with COVID-19 symptoms. By increasing staff hours and changing EMS protocol, Tehran became better suited to deliver medical care to a larger number of patients while minimizing unnecessary exposure to COVID-19. The healthcare system in Iran must continue surveillance of its crisis plans for prehospital and hospital services in order to continue optimizing its response. Because the pandemic continues, all hospitals need to estimate their capacity and predict the amount of resources and number of staff required to fight this pandemic. Lessons learned from this pandemic will help us to guide specific management and disaster planning for future healthcare crises.

## LIMITATIONS

This study is limited in its scope as it focuses on the EMS response in Tehran, Iran. While COVID-19 is causing a worldwide health crisis, these data are not, per se, generalizable. It is likely that other health systems have faced similar challenges with an increase in the number of EMS dispatches and calls. This study can provide guidance for other EMS units fighting this pandemic. We did not collect any outcome data regarding the EMS dispatches.

## CONCLUSION

Tehran’s medical system saw an increase in the number of patients with COVID-19 symptoms soon after the beginning of the outbreak. We found a 347% increase in EMS calls and a 21% increase in EMS dispatches. We increased the number of EMS personnel in dispatch by up to 140%, but did not change the number of first responders. The EMS in Tehran changed the way it delivered care by increasing the number of personnel, reducing time off between shifts, and increasing overtime hours, which helped to ease the burden of the pandemic. There has been continued in-service education during this outbreak. We hope that the COVID-19 pandemic is limited, but we should continue to consider better approaches that our patients and providers deserve. Working to mitigate this crisis will help us better prepare for future inevitable pandemics.

## Figures and Tables

**Table 1:** Emergency medical services dispatches and calls before and after the beginning of the COVID-19 outbreak.

Study variable	Pre-outbreak	Post-outbreak	Percent change	P-value
EMS calls N (% of total)	355,241 (18.2)	1,589,346 (81.8)	347	<0.001
EMS missions N (% of total)	82,282 (45.1)	99,926 (54.9)	21	<0.001
Time of call (min)				
Median (Q1, Q3)	0.7 (0.3, 2.1)	0.50 (0.32, 1.3)	−29	<0.001
Mean±SD	1.3 ± 1.4	1.1 ± 1.3	−15	<0.001
Time waiting on hold (sec)				
Median (Q1, Q3)	7.0 (7.0, 7.0)	7.0 (7.0, 7.0)	0	1.00
Mean±SD	10.6 ± 12.7	9.8 ± 11.8	−8	<0.001

* *EMS*, emergency medical services; *Q1*, 25th percentile; *Q3*, 75th percentile; *SD*, standard deviation.

**Table 2:** Comparison of chief complaints and vital signs in emergency medical services dispatches before and after the start of the COVID-19 outbreak.

Study variable	Pre-outbreak	Post-outbreak	Percent change	P-value
Chief complaint				
Trauma	6993 (11.4)	3282 (4.3)	−53	<0.001
Motor vehicle accident	5358 (8.7)	3699 (4.8)	−31	<0.001
Fever	578 (0.9)	1796 (2.3)	211	<0.001
Respiratory complaints	3299 (5.4)	11,371 (14.7)	245	<0.001
Cardiopulmonary arrest	1257 (2.1)	1492 (1.9)	19	0.128
Cardiovascular complaints	9122 (14.9)	9530 (12.3)	5	<0.001
Gynecologic emergencies	145 (0.2)	135 (0.2)	−7	0.012
Gastrointestinal complaints	3987 (6.5)	4371 (5.7)	10	<0.001
Neurologic complaints	8316 (13.5)	8147 (10.6)	−2	<0.001
Psychiatric complaints	5545 (9.0)	4057 (5.3)	−27	<0.001
Diabetic emergencies	14,064 (22.9)	20,295 (26.3)	44	<0.001
Toxicity	1252 (2.0)	614 (0.8)	−51	<0.001
Others	1524 (2.5)	8470 (11.0)	456	<0.001
TOTAL	61,440 (100)	77,259 (100)	26	<0.001

* *EMS*, emergency medical services.
